# Hydrological Conditions Drive Colonial Waterbird Breeding Dynamics in Monsoon‐Dependent Wetlands of Southern India

**DOI:** 10.1002/ece3.74567

**Published:** 2026-10-21

**Authors:** Alex Jacob, Govindan Veeraswami Gopi

**Affiliations:** ^1^ Department of Endangered Species Management Wildlife Institute of India Dehradun Uttarakhand India; ^2^ Academy of Scientific and Innovative Research (AcSIR) Ghaziabad Uttar Pradesh India

**Keywords:** breeding ecology, climatic variability, colonial nesting waterbirds, environmental drivers, wetland inundation

## Abstract

Wetland ecosystems are highly sensitive to environmental variability, but the relative importance of hydrological and climatic drivers of colonial waterbird breeding is poorly understood in landscapes that depend on the tropical monsoon. We investigated the influence of wetland inundation and climatic conditions on the occurrence of nesting, colony abundance and species richness of colonial nesting waterbirds across nine wetlands in Tamil Nadu, India, over three breeding seasons (2022–2023 to 2024–2025). Breeding responses were quantified through systematic field surveys and related to wetland water extent derived from Sentinel‐2 imagery and climatic variables obtained from the ERA5 reanalysis dataset. We measured the effects of water extent, temperature, precipitation and wind speed with generalised linear mixed models. A total of 16 colonial nesting waterbird species were recorded. Environmental conditions during the 30‐day period preceding surveys consistently provided the strongest explanation of breeding responses. Water extent emerged as the dominant predictor of nesting occurrence, colony abundance and species richness. The nesting probability increased significantly with water extent (*β* = 1.732, *p* < 0.001), whereas colony abundance (*β* = 0.612, *p* < 0.001) and species richness (*β* = 0.275, *p* < 0.001) increased with greater inundation. Maximum temperature negatively affected colony abundance and species richness, whereas precipitation had weaker negative effects and wind speed had no significant effect. Our findings indicate that the main driver of colonial waterbird breeding dynamics in monsoon‐dependent wetlands is wetland inundation, while climatic factors exert secondary effects on colony performance and diversity. Securing suitable hydrological conditions will be essential to the conservation of colonial waterbird populations in the face of increasing climatic variability.

## Graphical abstract

Colonial waterbirds depend on wetlands for breeding, yet the relative importance of hydrological conditions and short‐term climatic variability remains poorly understood in monsoon‐dependent systems. Using three years of data from nine wetlands in southern India, we found that wetland water extent was the strongest predictor of nesting arrival, colony abundance and species richness, while climatic variables had weaker effects and environmental conditions during the preceding 30 days best explained breeding responses. Our results reveal that hydrological conditions are the most important driver of colonial waterbird breeding dynamics, and highlight the importance of wetland inundation maintenance for conservation and management.

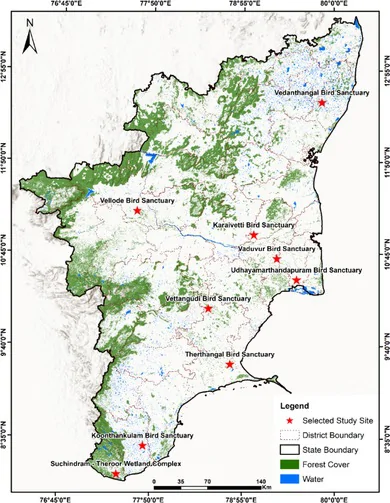

## 1 Introduction

Wetlands are ecologically important and highly productive ecosystems that support a diverse array of species relative to other habitats. They provide important habitat for fish, amphibians, invertebrates, reptiles and birds and ecosystem services including water purification, flood control, nutrient cycling and carbon storage (Zedler and Kercher 2005). Waterbirds are strongly associated with wetlands because their essential life processes, such as nesting, foraging and roosting, are highly dependent on wetland characteristics, especially hydrological conditions. Therefore, changes in wetland water levels, the stability of water periods and prey availability can quickly impact the waterbird populations (Rajpar and Zakaria 2011; Chastant and Gawlik 2018).

Climatic variables are a key regulator of wetland structure and function (Salimi et al. 2021). Temperature affects evaporation rates, primary productivity and metabolic demands within wetland ecosystems, while precipitation regulates water depth, flooding extent and wetland connectivity (Zhang et al. 2025). In monsoon‐driven regions, the seasonal flooding cycles, which have a crucial role in shaping food webs and determining habitat suitability, depend on the pattern and duration of rainfall (Pereira et al. 2021). However, wetlands are inherently vulnerable to weather‐related fluctuations. Increased evapotranspiration can shorten the hydroperiod, while changes in the timing or amount of rainfall may lead to longer periods of dry conditions or unstable flooding (Kovářová and Pokorný 2010; Balerna et al. 2023). Thus, climatic variability can influence changes in wetland hydrology, which subsequently influence the ecological conditions experienced by wetland‐dependent species. Thus, climatic variability can translate into changes in wetland hydrology, which subsequently influence the ecological conditions experienced by wetland‐dependent species.

Hydrological variability provides the ecological link between climatic conditions and waterbird breeding responses. The extent of flooding and hydroperiod affect wetland productivity through effects on nutrient cycling, primary production and the development of aquatic food webs, with implications for abundance and accessibility of fish and aquatic invertebrates that are important prey resources for waterbirds (Frost et al. 2023; Byrne et al. 2025). The efficiency with which birds can exploit these prey resources is further influenced by water depth and the spatial distribution of inundated habitats. Therefore, hydrological conditions are particularly important when assessing habitat quality. Rainfall may therefore influence breeding indirectly through its effects on wetland inundation, hydroperiod and associated foraging conditions, while hydrological conditions also reflect watershed characteristics and human regulation of water availability. The extent and persistence of wetlands in terms of foraging conditions to support incubation and chick‐rearing will likely govern breeding decisions, while continued hydrological suitability will influence colony performance and reproductive success throughout the breeding season (Beerens et al. 2011; Essian et al. 2022, 2025).

Colonial nesting waterbirds constitute a unique ecological guild within taxa dependent on wetlands. These birds breed in large groups and often form large heronries in or around wetlands, utilising trees or plants growing above water. This group includes herons, egrets, cormorants, darters, ibises, storks, spoonbills and other associated species. In India, 26 species of colonial nesting waterbirds have been recorded (Subramanya 1996), of which 19 species have been recorded breeding in Tamil Nadu (Frank et al. 2021). Breeding in colonies provides benefits such as increased detection of predators and transfer of information about food resources (Kopachena 1991), but can also lead to increased vulnerability to environmental conditions because many individuals will nest in the same location (Alexander 1974). Thus, the continued existence of breeding colonies is dependent on hydrologic conditions, suitable nesting substrates and reliable prey resources (Frederick and Collopy 1989; Kingsford and Johnson 1998; Gawlik 2002).

Tamil Nadu supports an extensive and varied wetland network which plays a crucial role in sustaining colonial breeding waterbirds. The state has mapped 24,089 inland wetlands at a 1:50,000 scale (National Wetland Atlas 2009), including irrigation tanks, freshwater lakes, reservoirs and marshes. The wetland habitats of Tamil Nadu support extensive and varied nesting of colonial nesting waterbirds. Tamil Nadu has 20 Ramsar sites, wetlands of international importance, making it the state with the most Ramsar‐designated wetlands in India. Many of these wetlands are strongly influenced by the northeast monsoon; therefore, a lack of seasonal rainfall can dry out wetlands, leading to the absence of breeding colonies in certain years (Jabaraj and Gopi 2020). Therefore, these wetlands are important breeding areas for colonial waterbirds in the region. A recent study across Tamil Nadu found 101 active heronries (Frank et al. 2021), underscoring the importance of the region as a breeding stronghold in southern India. Several wetlands are nationally and internationally recognised for their ecological value, yet many are hydrologically dependent on monsoon rainfall and water releases from distant reservoirs or canal systems.

Globally, a considerable amount of research suggests that the breeding behaviours of colonial waterbirds are influenced by changes in climate and water availability (Celik 2026). In particular, higher temperatures have been linked to reduced breeding success and increased chick mortality, which is likely due to thermal stress (Van de Ven et al. 2019; Conradie et al. 2019). Furthermore, the extent of rainfall and flooding significantly affects colony establishment, breeding population sizes and the availability of suitable foraging habitats (Kingsford and Johnson 1998; Bino et al. 2014). Rapid changes in water levels may lead to nest abandonment or even abandonment of entire colonies (Torres et al. 2010; Leslie 2001). Hydrological instability has also been associated with inter‐annual variations in the abundance of breeding populations (Leberg et al. 2007). Studies on species such as storks, ibises and herons in India have also shown the importance of rainfall, water depth and prey availability in determining breeding patterns (Tiwary and Urfi 2016; Ahmed and Urfi 2024). However, the magnitude and nature of these relationships are often variable across regions depending on hydrological regimes, climatic conditions and water management strategies.

Although Tamil Nadu is home to 19 colonial nesting waterbird species and more than 100 heronries, quantitative multi‐site assessments on the influence of short‐term climatic variability on breeding dynamics are lacking. Previous studies have focused on isolated wetlands or specific species, with few studies evaluating both nesting arrival (colony initiation) and colony abundance at multiple locations and seasonal periods simultaneously (Frank et al. 2022). Moreover, no comprehensive analysis of the influence of temperature, precipitation and wind patterns on the pre‐breeding phase in monsoon‐dependent wetlands in southern India has been conducted.

This study attempts to fill this knowledge gap by investigating the relative importance of hydrological and climatic conditions on colonial nesting waterbirds at nine wetlands in Tamil Nadu over three consecutive breeding seasons (2022–2023 to 2024–2025). Specifically, we: (1) quantified inter‐annual variation in nesting occurrence and species richness; (2) compared alternative temporal windows (30‐, 60‐ and 90‐day periods prior to surveys) to determine the environmental time scale to which breeding responses were most strongly associated, recognising that environmental conditions prior to breeding may affect birds at different temporal scales and may reflect recent versus accumulated climatic effects; and (3) investigated the effects of wetland water extent, maximum temperature, precipitation and wind speed on nesting arrival, colony abundance and species richness using generalised linear mixed models. Combining systematic field surveys, satellite‐based estimates of wetland inundation and spatially consistent climate data, this study provides a regional scale assessment of the environmental drivers of colonial waterbird breeding dynamics in the monsoon‐dependent wetlands of southern India.

We predicted that colony establishment, abundance and species richness would be positively associated with wetland inundation because surface water provides suitable nesting and foraging conditions for colonial waterbirds. We also hypothesised that increased temperatures would negatively affect breeding populations through increased thermal stress and reduced habitat quality, that precipitation would have indirect positive effects through its influence on wetland hydrology and that wind speed would have a negative effect on breeding activity through reduced flight efficiency and nest stability.

## 2 Materials and Methods

### 2.1 Study Area

The study was conducted in nine wetlands in Tamil Nadu, southern India (Figure 1). Tamil Nadu has a tropical monsoon climate, with the majority of the annual rainfall occurring during the northeast monsoon season between October and December (Subramanya 2005). Most of the wetlands selected for this study are in the eastern and southern parts of the state, except Vellode Bird Sanctuary, which is located in the western region of Tamil Nadu (Table 1). Most of the wetlands are small to medium in size with their area ranging from 29.3 to 112.6 ha. In contrast, the Karaivetti Bird Sanctuary is much larger, covering an area of 453.7 ha, which is considerably larger than the other sites.

**FIGURE 1:**
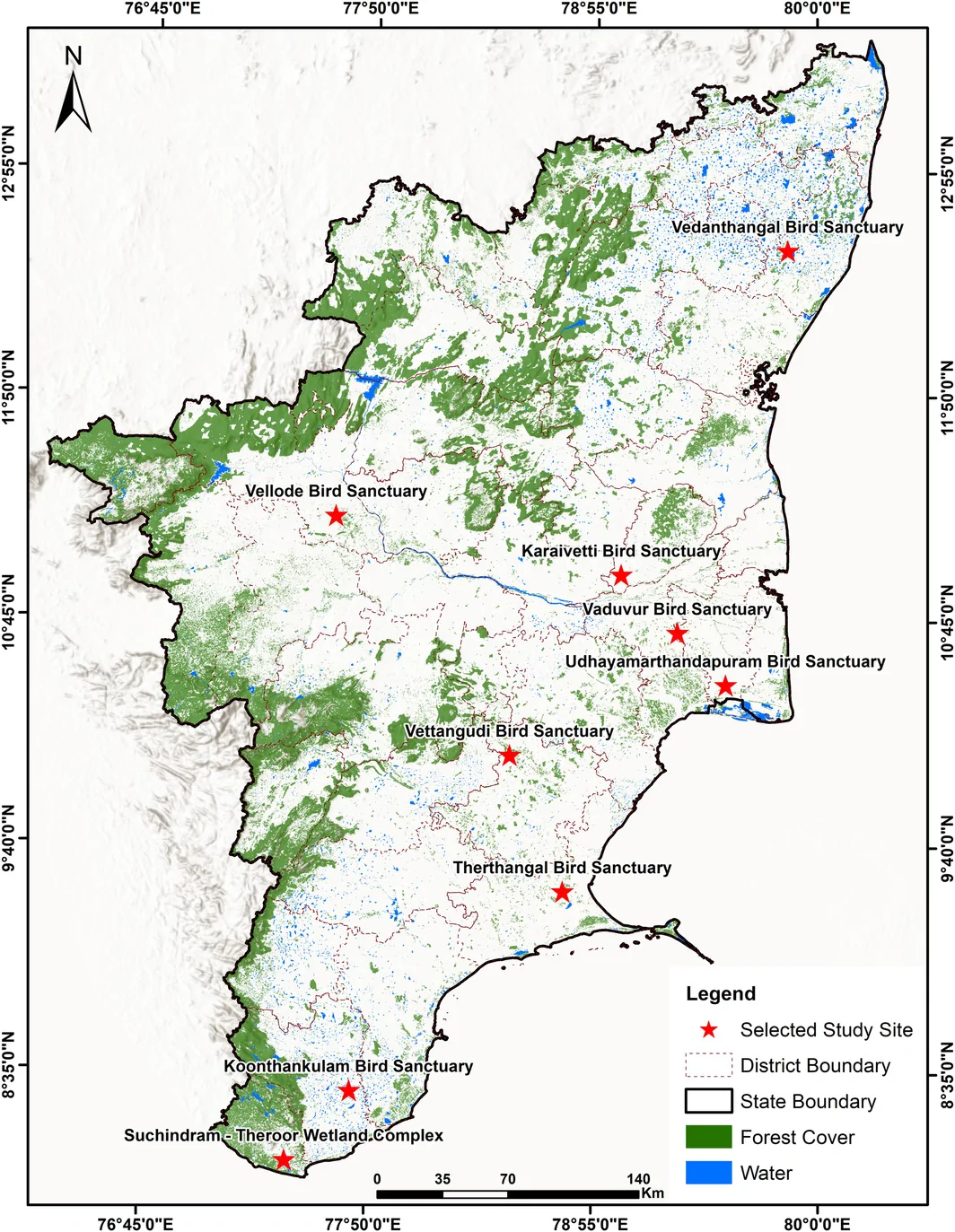
Study area map of selected wetlands in Tamil Nadu, India. The map shows the nine study sites (red stars) across the state. State and district boundaries are indicated, along with forest cover (green) and water bodies (blue).

**TABLE 1:** Geographic location and key characteristics of the nine study wetlands in Tamil Nadu.

Site	District	Latitude (°N)	Longitude (°E)	Area (ha)	Ramsar status
Vedanthangal Bird Sanctuary	Chengalpattu	12.5452	79.8557	40.3	Yes
Vellode Bird Sanctuary	Erode	11.2515	77.6520	77.2	Yes
Karaivetti Bird Sanctuary	Ariyalur	10.9783	79.0454	453.7	Yes
Vaduvur Bird Sanctuary	Thiruvarur	10.7003	79.3204	112.6	Yes
Udhayamarthandapuram Bird Sanctuary	Thiruvarur	10.4498	79.5549	43.8	Yes
Vettangudi Bird Sanctuary	Sivagangai	10.1087	78.5099	38.4	No
Therthangal Bird Sanctuary	Ramanathapuram	9.4559	78.7710	29.3	Yes
Koonthankulam Bird Sanctuary	Tirunelveli	8.4962	77.7573	72.0	Yes
Suchindram–Theroor Wetland Complex	Kanyakumari	8.1608	77.4521	94.2	Yes

All the study sites are artificial wetlands, constructed mainly for water storage and agricultural irrigation. Although they share a similar management purpose, the wetlands vary in their structure and hydrological features. Karaivetti Bird Sanctuary is a large, inland freshwater wetland, while Vaduvur Bird Sanctuary and the Suchindram Theroor Wetland Complex are larger reservoir‐type wetlands. Therthangal Bird Sanctuary is a marshy wetland system, whereas the remaining sites are typical irrigation tanks embedded within agricultural landscapes. Most of these wetlands are either rain‐fed or supplemented by inflows from canal networks and nearby river systems. All these wetlands are found in agricultural areas and are used for irrigation purposes, especially for paddy cultivation. These irrigation tanks and reservoirs, which are essential to Tamil Nadu's traditional water management, also serve as important nesting sites for colonial waterbirds.

The nine study sites were selected from wetlands known to support important colonial waterbirds' breeding colonies and where repeated field monitoring was feasible. The selected wetlands also represented different rainfall and climatic conditions, as well as different wetland types and hydrological settings across Tamil Nadu. Eight of the nine study wetlands are Ramsar sites, reflecting their recognised ecological importance for waterbirds. Wetland pairs that were close together were about 40–80 km apart, while the other sites were more than 120 km apart.

### 2.2 Bird Surveys

Bird surveys were conducted over three consecutive breeding seasons: 2022–2023, 2023–2024 and 2024–2025. The 2022–2023 season surveys were completed from October 2022 to June 2023. The 2023–2024 season was surveyed from August 2023 to June 2024, and the 2024–2025 season from July 2024 to May 2025.

Each wetland was visited at approximately monthly intervals during the breeding season, and bird counts were conducted during every visit. All study sites supported both nesting and roosting colonial nesting waterbirds. As roosting birds arrive at nesting sites in the evening and depart early in the morning, counts were conducted after roosting birds had left the colonies to avoid overestimation of breeding populations.

Surveys were performed between 7 and 8 a.m. from fixed vantage points selected to provide complete or near‐complete visual coverage of nesting colonies. Existing watchtowers within wetlands served as the primary vantage points whenever available and were supplemented by additional observation points when required. The number of vantage points varied among wetlands depending on colony size, colony distribution and visibility of nesting areas rather than wetland area alone. Wetlands supporting large or spatially dispersed colonies required multiple vantage points, whereas sites with compact colonies could be surveyed from a single location. The same set of vantage points was used consistently within each wetland across survey visits whenever possible. Counts were conducted at a consistent time of day because colonial nesting waterbirds spend substantial periods away from nests during daylight hours for foraging or collecting nesting material, resulting in fluctuations in the number of adults present in the colonies.

The standard total count method for colonial nesting waterbirds was followed (Bibby 2000). At the onset of each breeding season, only individuals that had initiated nest building or formed breeding pairs were included in counts. During subsequent visits, both adults attending nests and chicks present in nests were recorded. All surveys were conducted by a single observer to maintain consistency in detection and counting procedures across sites and years. Birds were identified and counted using Nikon Monarch 7 binoculars (8 × 42) and a Sony RX10 IV camera, which was also used to verify counts and species identification when necessary.

### 2.3 Climate Data

Climate data were obtained from the ERA5 reanalysis dataset maintained and provided by the Copernicus Climate Change Service (Copernicus Climate Change Service 2018). This dataset is a combination of satellite observations, ground‐based measurements and numerical weather prediction model outputs to generate spatially and temporally consistent climate information (Hersbach et al. 2020). Hourly data, in NetCDF format, were downloaded from the Climate Data Store at a spatial resolution of approximately 0.25° (~31 km) for Tamil Nadu, India (8.0°–14.5° N, 76.0°–80.5° E).

The following variables were extracted: total precipitation, 2‐m air temperature and 10‐m wind components (u and v). For each wetland, climatic variables were extracted from the nearest ERA5 grid cell based on site coordinates. Hourly data were aggregated to daily values prior to analysis. Daily maximum air temperature (°C) was derived from hourly 2‐m air temperature following conversion from Kelvin to Celsius. Daily cumulative precipitation (mm) was calculated from hourly total precipitation. Daily mean wind speed (m s^−1^) was calculated from the zonal (*u*; east–west) and meridional (*v*; north–south) wind components using vector magnitude (√(*u* ^2^ + *v* ^2^)).

To evaluate climatic influences on breeding dynamics, climatic variables were summarised over 30‐, 60‐ and 90‐day periods preceding each survey. Maximum temperature and wind speed were averaged across each temporal window, whereas precipitation was summed to obtain cumulative rainfall. Candidate models incorporating each temporal window were compared using Akaike's Information Criterion (AIC), and the best‐supported temporal scale was retained for subsequent analyses.

All data extraction, processing and aggregation were conducted in Python using the *xarray*, *numpy* and *pandas* libraries. Climatic predictors were standardised (mean = 0, SD = 1) prior to statistical analyses.

### 2.4 Wetland Hydrology

Wetland hydrological conditions were quantified using Sentinel‐2 Surface Reflectance Harmonised imagery (COPERNICUS/S2_SR_HARMONISED) at 10‐m spatial resolution. Images with cloud cover exceeding 60% were excluded. For each survey, a composite image was generated using all available Sentinel‐2 observations acquired during the 15‐day period preceding the survey date.

Surface water extent was assessed using the Modified Normalised Difference Water Index (MNDWI), calculated as (Green − SWIR)/(Green + SWIR), where Green and SWIR correspond to Sentinel‐2 Bands 3 and 11, respectively. Mean MNDWI composites were generated for each survey period and clipped to wetland boundaries.

Water pixels were classified using wetland‐specific MNDWI thresholds because spectral responses varied among wetlands owing to differences in water colour, aquatic vegetation, turbidity and surrounding land cover. For each wetland, candidate MNDWI threshold values were evaluated iteratively by comparing MNDWI values of individual pixels with the corresponding areas in contemporaneous true‐colour Sentinel‐2 imagery, with field observations of water conditions from repeated surveys across the three breeding seasons. The threshold was progressively adjusted through a trial‐and‐evaluation process until the resulting classification showed the best correspondence with visually identifiable open‐water areas. These visually delineated open‐water areas served as reference information during threshold selection.

### 2.5 Statistical Analysis

All statistical analyses were conducted in R version 4.3.3 (R Core Team 2024). Nesting arrival was analysed using generalised linear mixed models (GLMMs) with a binomial error distribution and logit link function. Arrival was treated as a binary response variable indicating the presence or absence of active nesting colonies during each survey.

Colony abundance was analysed using GLMMs with a negative binomial error distribution because abundance data exhibited substantial overdispersion relative to a Poisson distribution (Çelik and Durmuş 2020). Species richness was analysed using GLMMs with a Poisson error distribution. For all models, wetland identity and breeding season were included as random intercepts to account for repeated observations across sites and years. Variance inflation factors (VIFs) were used to assess multicollinearity among predictor variables and all VIF values < 2 indicated low collinearity among predictors.

Fixed effects included maximum temperature, cumulative precipitation, mean wind speed and water extent. Climatic variables summarised over 30‐, 60‐ and 90‐day periods preceding surveys were used as candidate predictors. Water extent (%) was included as a direct measure of wetland hydrological conditions. Continuous predictor variables were standardised (mean = 0, SD = 1) prior to analysis to facilitate comparison of effect sizes and improve model convergence.

Competing models based on the three temporal windows were compared using Akaike's Information Criterion (AIC), and the temporal window receiving the strongest support was retained for subsequent analyses. Additional model comparisons were performed to evaluate the relative contributions of hydrological and climatic predictors by comparing water extent‐only models, water extent plus temperature models and full models containing all environmental variables.

Binomial GLMMs were fitted using the package lme4, whereas negative binomial and Poisson GLMMs were fitted using glmmTMB. Model assumptions and goodness‐of‐fit were evaluated using simulation‐based residual diagnostics implemented in the DHARMa package.

## 3 Results

### 3.1 Nesting Occurrence and Species Richness

Colonial waterbird nesting was recorded at all nine wetlands in atleast one of the three breeding seasons (2022–2023 to 2024–2025). Six of the nine wetlands supported nesting in all surveyed years. Nesting was absent at Koonthankulam and Therthangal during the 2022–2023 season and at Vettangudi during the 2023–2024 season. Although nesting was present at Karaivetti during 2023–2024, systematic abundance data were not collected at this site during that particular breeding season (Table 2).

**TABLE 2:** Seasonal occurrence of colonial waterbird nesting across nine wetlands of Tamil Nadu (2022–2023 to 2024–2025).

Site	2022–2023	2023–2024	2024–2025
Vedanthangal Bird Sanctuary	Nesting present	Nesting present	Nesting present
Vellode Bird Sanctuary	Nesting present	Nesting present	Nesting present
Karaivetti Bird Sanctuary	Nesting present	Data not collected	Nesting present
Vaduvur Bird Sanctuary	Nesting present	Nesting present	Nesting present
Udayamarthandapuram Bird Sanctuary	Nesting present	Nesting present	Nesting present
Vettangudi Bird Sanctuary	Nesting present	Nesting absent	Nesting present
Therthangal Bird Sanctuary	Nesting absent	Nesting present	Nesting present
Koonthankulam Bird Sanctuary	Nesting absent	Nesting present	Nesting present
Suchindram‐Theroor Wetland Complex	Nesting present	Nesting present	Nesting present

A total of 16 colonial nesting waterbird species were recorded across the nine wetlands during the study period. Species richness varied among wetlands and breeding seasons (Table 3). Species richness was relatively stable in Vedanthangal and Vaduvur, whereas Vellode showed a marked decline in species richness from 10 species in 2022–2023 to 6 species in 2023–2024 and 5 species in 2024–2025. Udhayamarthandapuram showed relatively minor interannual variation whereas Therthangal, Vettangudi and Koonthankulam showed substantial temporal fluctuations associated with presence or absence of breeding colonies. Species‐specific abundance patterns across wetlands and breeding seasons are shown in Figure 2.

**TABLE 3:** Inter‐annual variation in nesting species richness of colonial waterbirds across wetlands (2022–2025).

Site	2022–2023	2023–2024	2024–2025	Species richness in three years
Vedanthangal Bird Sanctuary	12	12	12	13
Vellode Bird Sanctuary	10	6	5	10
Karaivetti Bird Sanctuary	9	—	10	10
Vaduvur Bird Sanctuary	9	9	9	11
Udayamarthandapuram Bird Sanctuary	9	10	10	10
Vettangudi Bird Sanctuary	6	0	9	9
Therthangal Bird Sanctuary	0	12	12	12
Koonthankulam Bird Sanctuary	0	6	6	7
Suchindram‐Theroor Wetland Complex	7	6	6	8

**FIGURE 2:**
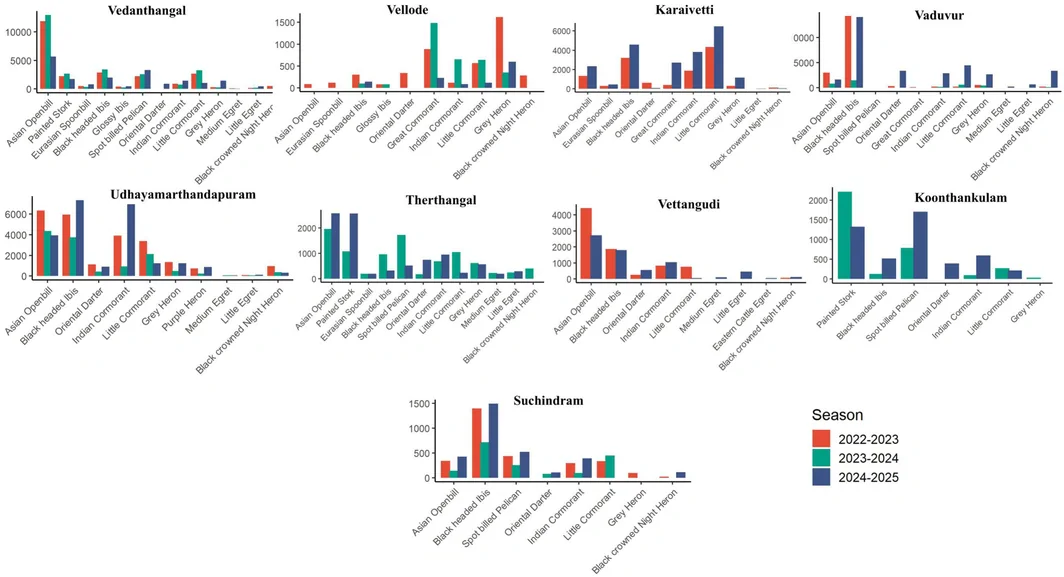
Bar plots showing species‐wise abundance of colonial nesting waterbirds across nine study sites during three breeding seasons (2022–2023, 2023–2024 and 2024–2025).

### 3.2 Temporal Scale Selection

Climatic variables summarised over 30‐, 60‐ and 90‐day periods preceding surveys were evaluated to determine the temporal scale most strongly associated with breeding responses. For all response variables, models based on climatic conditions during the 30‐day period received the strongest support (Table 4). Models based on 60‐ and 90‐day climatic windows had higher AIC values than the 30‐day models, indicating comparatively weaker support.

**TABLE 4:** Comparison of alternative climatic temporal windows used to model colonial waterbird breeding responses.

Response variable	Temporal window	AIC	ΔAIC
Nesting arrival	30‐day	138.56	0.00
	60‐day	144.34	5.78
	90‐day	145.41	6.84
Colony abundance	30‐day	3718.21	0.00
	60‐day	3724.67	6.46
	90‐day	3725.42	7.21
Species richness	30‐day	1280.02	0.00
	60‐day	1288.38	8.36
	90‐day	1286.48	6.46

Consequently, the 30‐day temporal window was retained for subsequent analyses of nesting arrival, colony abundance and species richness.

### 3.3 Drivers of Nesting Arrival

The best‐supported binomial generalised linear mixed model included maximum temperature, cumulative precipitation, mean wind speed and water extent as fixed effects, with wetland identity and breeding season included as random intercepts (Table 5).

**TABLE 5:** Fixed‐effect parameter estimates from the binomial generalised linear mixed model assessing environmental influences on colonial waterbird nesting arrival probability.

Predictor	Estimate (*β*)	SE	*z*	*p*
Intercept	3.453	0.932	3.704	< 0.001
*T*_max_ (30‐day)	0.059	0.337	0.175	0.861
Precipitation (30‐day)	−0.789	0.503	−1.569	0.117
Wind speed (30‐day)	−0.254	0.357	−0.713	0.476
Water extent	1.732	0.383	4.524	< 0.001

*Note:* Continuous predictors were standardised (mean = 0, SD = 1). Site and breeding season were included as random intercepts.

Water extent exerted a strong positive effect on nesting arrival probability (*β* = 1.732 ± 0.383 SE, *z* = 4.52, *p* < 0.001). In contrast, maximum temperature, precipitation and wind speed showed no significant associations with colony establishment (Table 5). The odds of colony establishment increased approximately 5.7‐fold for each one‐standard‐deviation increase in water extent.

Model predictions indicated that nesting probability increased rapidly with increasing wetland inundation (Figure 3). Predicted nesting probability increased from approximately 0.55 at very low levels of inundation to nearly 1.0 under highly inundated conditions.

**FIGURE 3:**
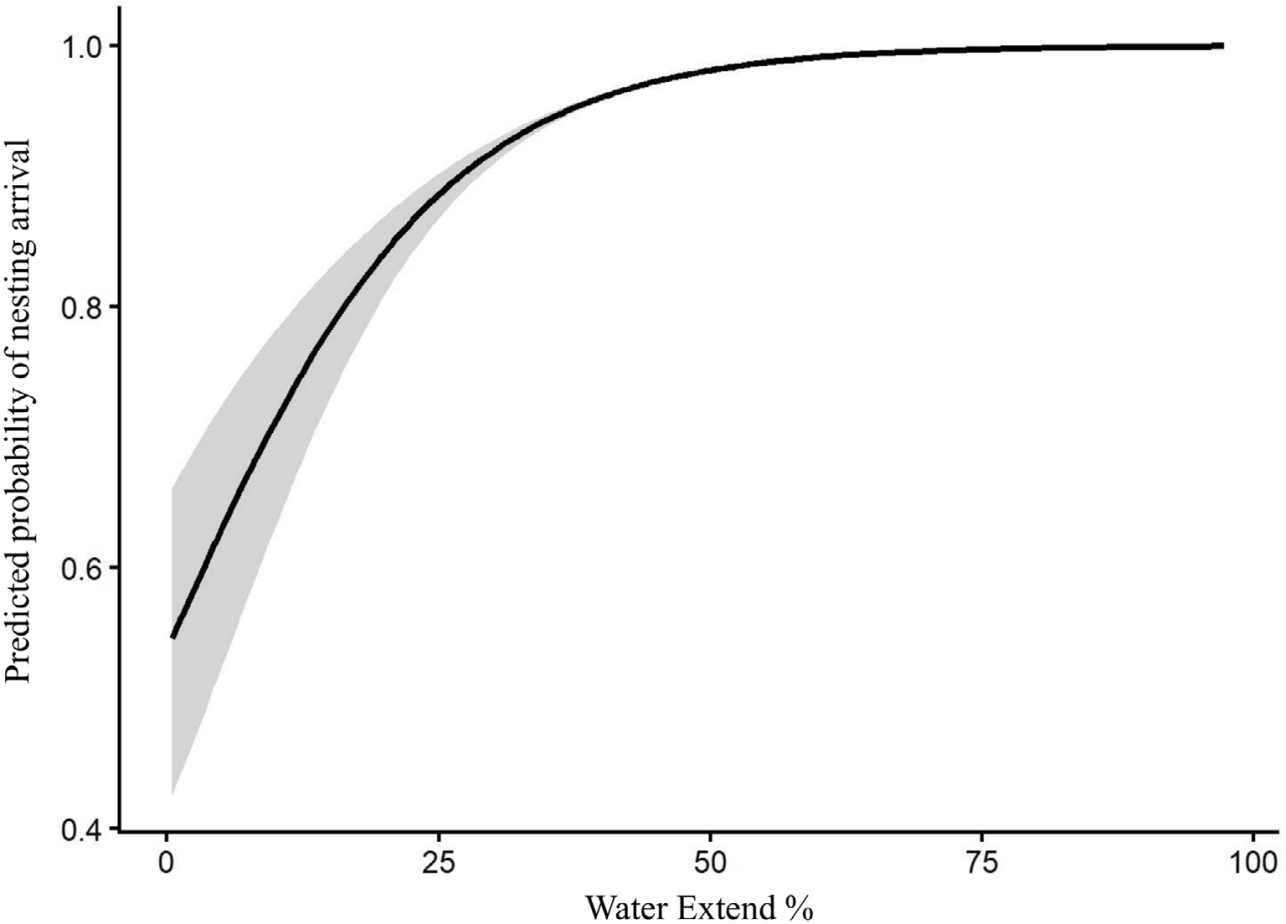
Predicted probability of colonial waterbird nesting arrival in relation to wetland water extent. Predictions were derived from the binomial generalised linear mixed model. Shaded areas represent 95% confidence intervals.

### 3.4 Drivers of Colony Abundance

Colony abundance was analysed using a negative binomial generalised linear mixed model including maximum temperature, cumulative precipitation, mean wind speed and water extent as fixed effects, with wetland identity and breeding season included as random intercepts (Table 6).

**TABLE 6:** Fixed‐effect parameter estimates from the negative binomial generalised linear mixed model assessing environmental influences on colonial waterbird abundance.

Predictor	Estimate (*β*)	SE	*z*	*p*
Intercept	6.488	0.354	18.321	< 0.001
*T*_max_ (30‐day)	−0.414	0.118	−3.511	< 0.001
Precipitation (30‐day)	−0.523	0.176	−2.973	0.003
Wind speed (30‐day)	−0.195	0.118	−1.652	0.099
Water extent	0.612	0.136	4.516	< 0.001

*Note:* Continuous predictors were standardised (mean = 0, SD = 1). Site and breeding season were included as random intercepts.

Water extent exerted a significant positive effect on colony abundance (*β* = 0.612 ± 0.136 SE, *z* = 4.52, *p* < 0.001), whereas maximum temperature showed a significant negative effect (*β* = −0.414 ± 0.118 SE, *z* = −3.51, *p* < 0.001). Precipitation was also negatively associated with colony abundance (*β* = −0.523 ± 0.176 SE, *z* = −2.97, *p* = 0.003), while wind speed showed a weak negative effect that was not statistically significant (*p* = 0.099).

Model predictions indicated a strong positive association between wetland inundation and colony abundance (Figure 4). Predicted abundance increased steadily with increasing water extent, with highly inundated wetlands supporting substantially larger breeding colonies than wetlands with limited surface water.

**FIGURE 4:**
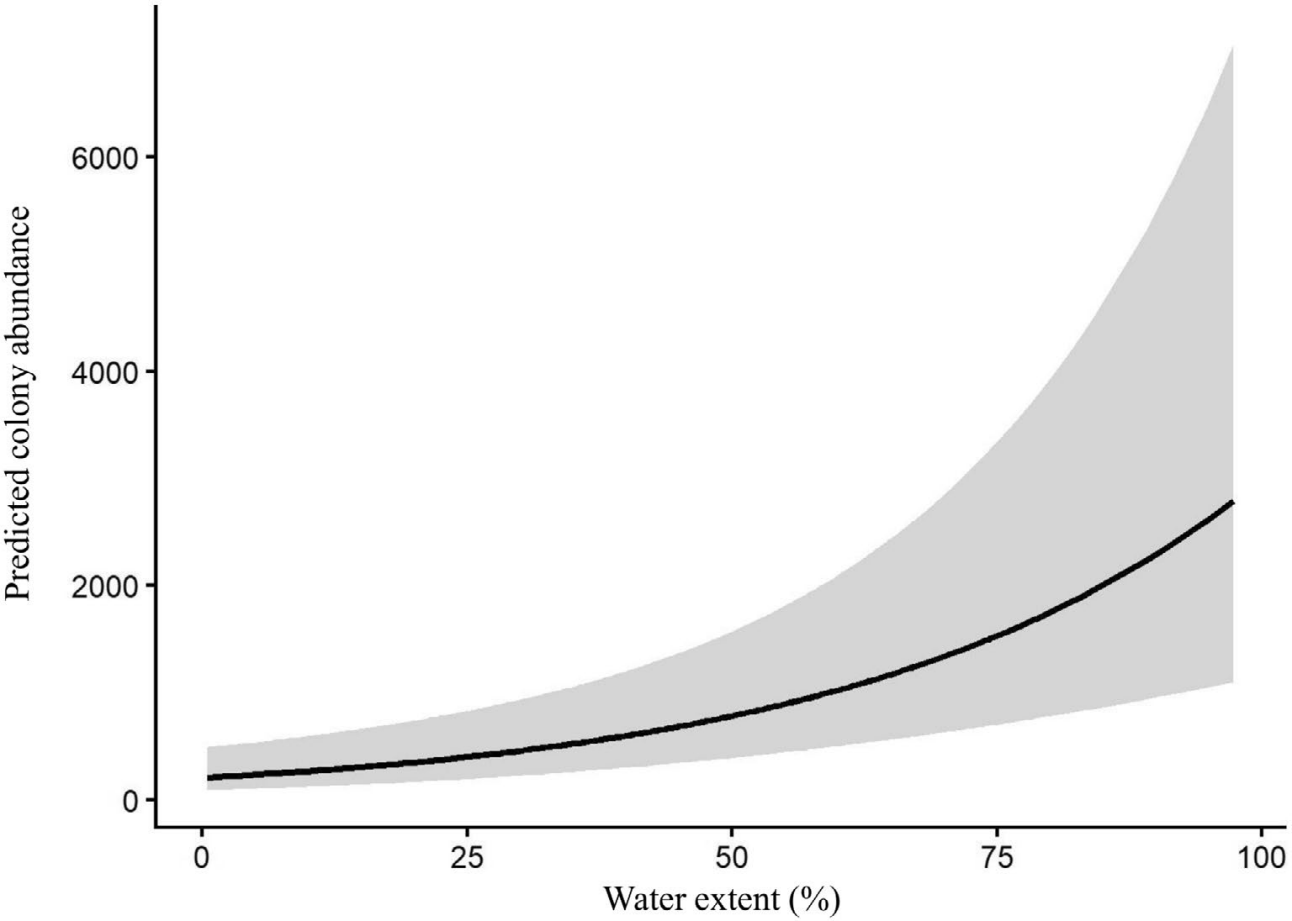
Predicted colony abundance of colonial nesting waterbirds in relation to wetland water extent. Predictions were derived from the negative binomial generalised linear mixed model. Shaded areas represent 95% confidence intervals.

Colony abundance also declined with increasing maximum temperature (Figure 5). Predicted abundance was highest under cooler conditions and decreased progressively with increasing temperature, indicating a negative relationship between thermal conditions and colony size.

**FIGURE 5:**
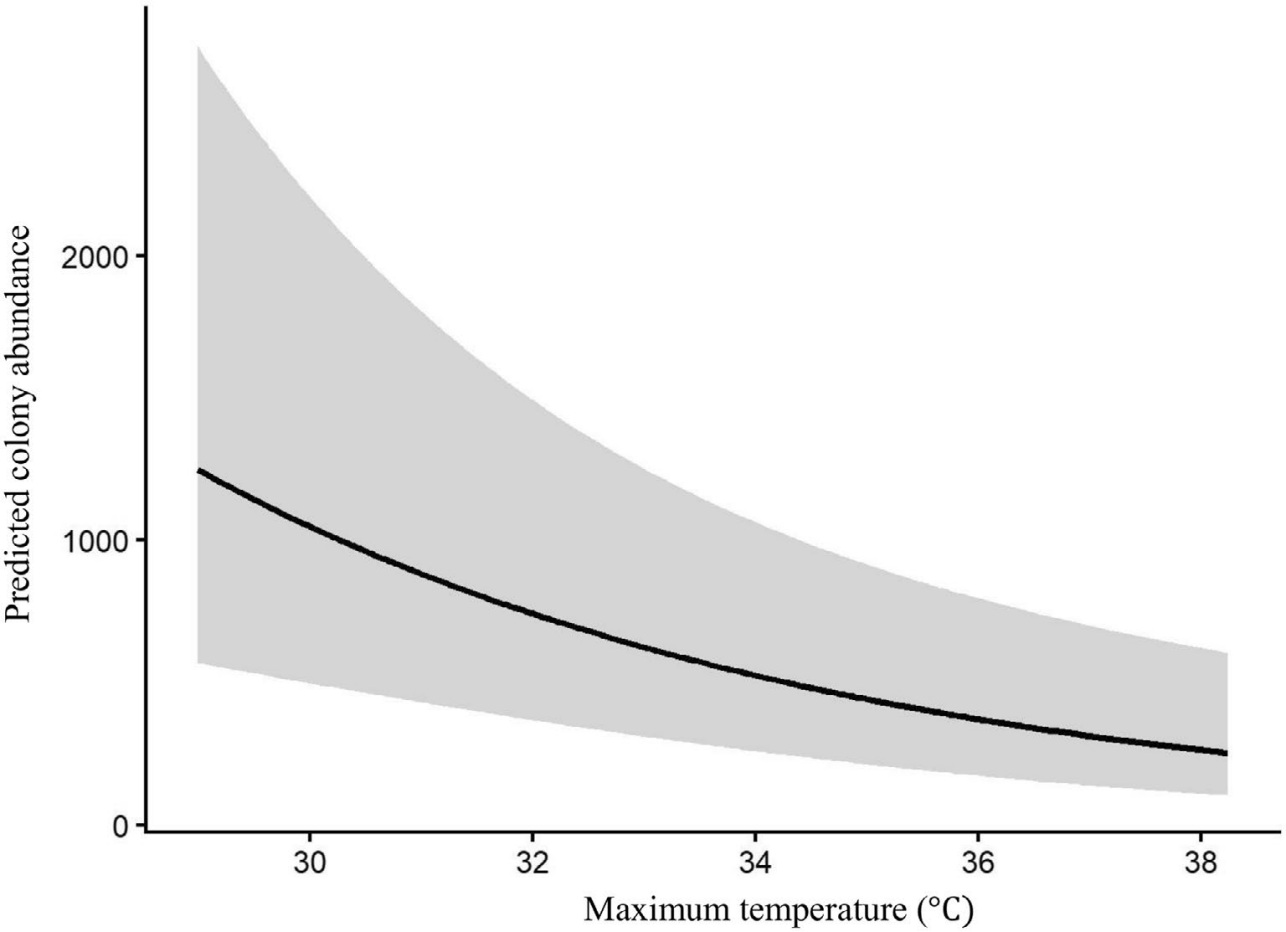
Predicted colony abundance of colonial nesting waterbirds in relation to 30‐day maximum temperature preceding surveys. Predictions were derived from the negative binomial generalised linear mixed model. Shaded areas represent 95% confidence intervals.

### 3.5 Drivers of Species Richness

Species richness was significantly associated with both hydrological and climatic conditions (Table 7). Water extent exerted a strong positive effect on species richness (*β* = 0.275 ± 0.032 SE, *z* = 8.71, *p* < 0.001), whereas maximum temperature showed a significant negative effect (*β* = −0.143 ± 0.032 SE, *z* = −4.44, *p* < 0.001). Precipitation was also negatively associated with species richness (*β* = −0.193 ± 0.064 SE, *z* = −3.04, *p* = 0.002), while wind speed showed no significant effect (*p* = 0.296).

**TABLE 7:** Fixed‐effect parameter estimates from the generalised linear mixed model assessing environmental influences on colonial waterbird species richness.

Predictor	Estimate (*β*)	SE	*z*	*p*
Intercept	1.623	0.139	11.716	< 0.001
*T*_max_ (30‐day)	−0.143	0.032	−4.443	< 0.001
Precipitation (30‐day)	−0.193	0.064	−3.039	0.002
Wind speed (30‐day)	−0.036	0.035	−1.046	0.296
Water extent	0.275	0.032	8.712	< 0.001

*Note:* Continuous predictors were standardised (mean = 0, SD = 1). Site and breeding season were included as random intercepts.

Water extent exhibited the strongest association with variation in species richness across wetlands and breeding seasons (Table 7). Model predictions indicated that species richness increased progressively with increasing water extent (Figure 6). Wetlands with greater inundation supported a higher number of nesting species, whereas species richness was lower under conditions of reduced water availability.

**FIGURE 6:**
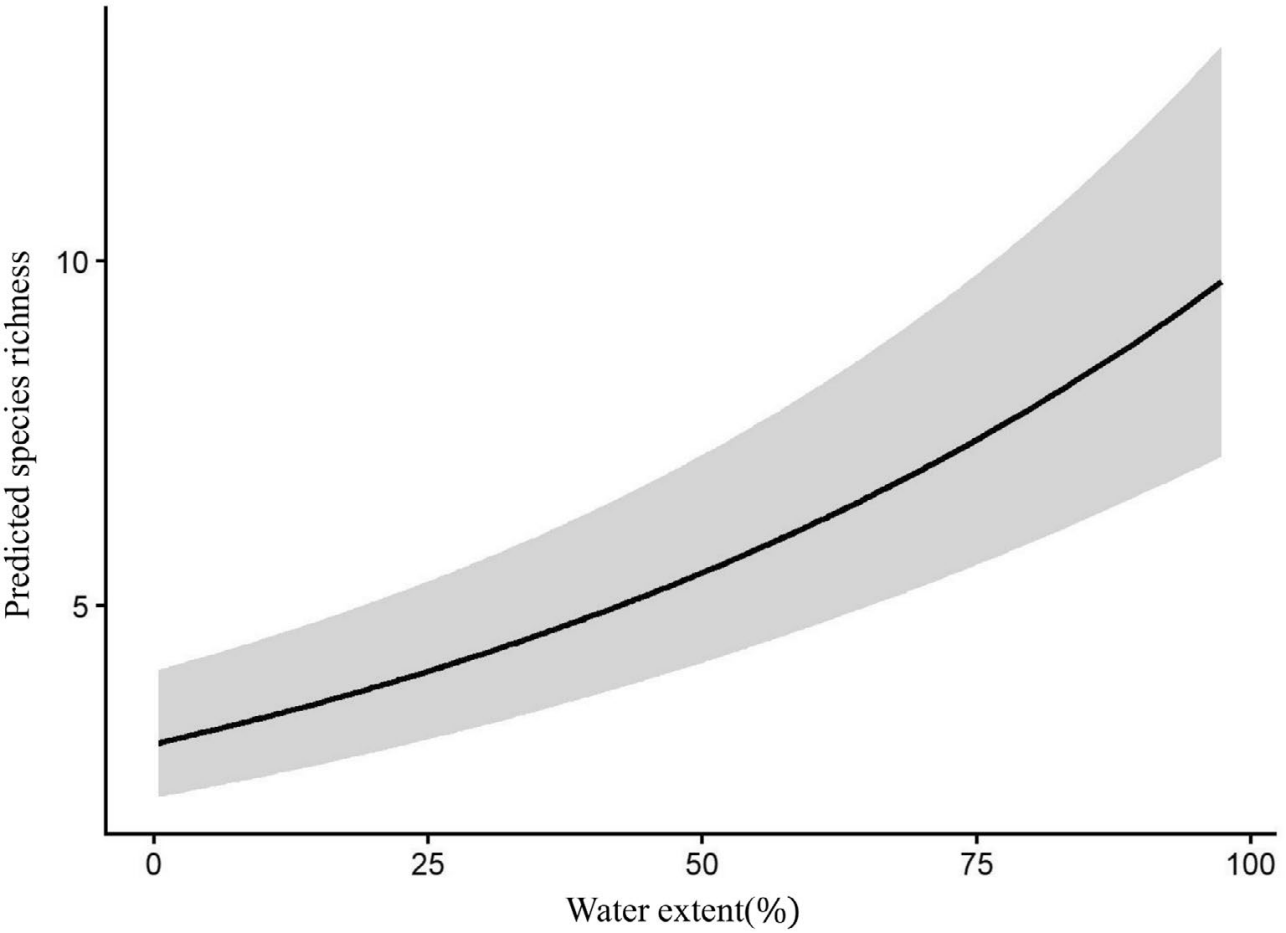
Predicted species richness of colonial nesting waterbirds in relation to wetland water extent. Predictions were derived from the generalised linear mixed model including water extent, maximum temperature, precipitation and wind speed as fixed effects and wetland identity and breeding season as random intercepts. Shaded areas represent 95% confidence intervals.

Species richness declined with increasing maximum temperature and precipitation, indicating that both hydrological conditions and short‐term climatic variation influenced the diversity of colonial nesting waterbirds across the study wetlands.

### 3.6 Relative Contributions of Hydrological and Climatic Predictors

Model comparisons showed that effects of hydrological conditions accounted for a large proportion of variation in both nesting arrival and colony abundance (Table 8). For nesting arrival, the water‐only model performed nearly as well as the full model (ΔAIC = 1.15), indicating that wetland inundation was the primary determinant of colony establishment. Inclusion of additional climatic variables resulted in only modest improvements in model fit.

**TABLE 8:** Comparison of alternative generalised linear mixed models evaluating the relative importance of hydrological (water extent) and climatic predictors.

Response variable	Model	AIC	ΔAIC
Nesting arrival	Full (Water + Temperature + Precipitation + Wind)	138.56	0.00
	Water only	139.72	1.15
	Water + Temperature	140.45	1.89
Colony abundance	Full (Water + Temperature + Precipitation + Wind)	3718.21	0.00
	Water + Temperature	3720.89	2.68
	Water only	3725.90	7.69

*Note:* Smaller AIC values indicate more support for the model. ΔAIC values were calculated relative to the best supported model for each response variable.

The model including water extent and temperature had stronger support than the water‐only model for colony abundance (ΔAIC = 5.01), indicating that temperature had explanatory power above hydrological conditions. The full model had the strongest overall support (AIC = 3718.21).

Together these results highlight that wetland inundation was the dominant driver of breeding activity, while climatic variables, particularly temperature, further influenced variation in colony abundance.

## 4 Discussion

This study shows that hydrological conditions are the main drivers of breeding dynamics of colonial nesting waterbirds across wetlands of Tamil Nadu. Wetland water extent was the strongest predictor of nesting arrival, colony abundance and species richness, indicating that the availability of inundated habitat governs both colony establishment and subsequent breeding activity. Climatic variables exerted secondary influences at the same time maximum temperature negatively affected colony abundance and species richness, while precipitation and wind speed showed weaker and less consistent effects. Models based on environmental conditions in the 30‐day period prior to the survey dates consistently performed better than models based on 60‐ and 90‐day periods, suggesting that colonial nesting waterbirds respond primarily to recent environmental conditions immediately prior to and during the breeding season. Together, these findings indicate that breeding responses are shaped by interactions between hydrological and climatic processes, with wetland inundation acting as the primary ecological filter influencing colony establishment and persistence.

Water extent was the strongest predictor of nesting arrival and exerted significant positive effects on both colony abundance and species richness. This finding is consistent with numerous studies showing that hydrological conditions are fundamental determinants of colonial waterbird breeding because flooding creates suitable foraging habitat, increases prey availability and reduces access by terrestrial predators (Frederick and Collopy 1989; Kingsford and Johnson 1998; Gawlik 2002; Kingsford et al. 2004). Water levels influence breeding directly by determining habitat availability and indirectly by affecting food resources and nesting security. Similar positive relationships between inundation and colonial waterbird breeding have been documented in the Dnestr Delta of Ukraine (Schogolev 1996), the Macquarie Marshes of Australia (Kingsford and Johnson 1998) and floodplain wetlands of Cameroon following hydrological restoration (Scholte 2006). Across these systems, colony establishment frequently occurs only when flooding creates sufficiently extensive and predictable habitat conditions to support breeding.

The strong influence of water extent on nesting arrival suggests that colonial nesting waterbirds are heavily reliant on hydrological cues to assess habitat suitability. Establishing a colony is a large reproductive effort and therefore requires assurance of the continued suitability of wetlands during incubation and chick rearing. Previous studies have shown that colony formation often depends on stable or predictably changing water levels rather than rainfall itself (Torres et al. 2010). Hydrological predictability may therefore be as important as hydrological extent in determining breeding decisions. The complete absence of nesting during some years at Koonthankulam, Therthangal and Vettangudi further illustrates the sensitivity of breeding activity to hydrological conditions.

Water extent also had a strong positive effect on species richness, suggesting that hydrological conditions influence not only whether birds breed but also the diversity of species that can take advantage of breeding sites. Larger inundated wetlands can provide a greater range of water depths, foraging opportunities, prey resources and microhabitats that can support species with differing ecological requirements (Torres et al. 2010). More broadly, wetland management studies consistently identify water availability, water depth and hydrological variability as among the most important determinants of waterbird diversity and habitat quality (Ma et al. 2010).

Despite the potential importance of precipitation as a driver of wetland productivity and waterbird breeding, rainfall had weak and inconsistent relationships with breeding responses in the current study. In contrast, direct measurements of wetland inundation were strong predictors of nesting activity. This apparent discrepancy is likely a reflection of the hydrological characteristics of many wetlands in Tamil Nadu. All the wetlands included in this study receive water from reservoirs, irrigation systems, canal networks and runoff from distant catchments. As a result, local rainfall may not reflect the hydrological conditions experienced at breeding sites. Similar patterns have been reported elsewhere. Torres et al. (2010) found that colonial waterbird breeding in Argentina responded more strongly to water levels than to local rainfall because hydrological conditions were controlled by rainfall occurring elsewhere within the watershed. Likewise, Chambers and Loyn (2006) demonstrated that waterbird abundance in Australian wetlands was often more strongly related to regional hydrological processes than to local rainfall. Water extent integrates the cumulative effects of rainfall, catchment processes, water management and evaporation and is therefore a more biologically meaningful measure of breeding habitat suitability than precipitation alone.

Maximum temperature was negatively associated with colony abundance and species richness, indicating that thermal conditions are important for breeding performance after colonies are established. Waterbird populations may differ in their sensitivity to climatic stressors, with temperature, precipitation and drought influencing population dynamics in species‐ and season‐specific ways (Bouregbi et al. 2025). Elevated temperatures can be metabolically demanding by increasing evaporative water loss and the energetic costs of thermoregulation. Under heat stress, birds often spend more time performing behaviours such as panting, wing‐spreading, gular fluttering, urohidrosis, and seeking shade, which may come at the expense of foraging and parental care (Van de Ven et al. 2019; Ahmed and Urfi 2024). High temperatures have been shown to reduce foraging efficiency, affect adult body condition, reduce chick provisioning rates and reduce breeding success in experimental and observational studies (Van de Ven et al. 2019, 2020). High temperatures may also have indirect effects on breeding performance through accelerated wetland drying, reduced hydroperiod length and changes in aquatic productivity that affect prey availability. The lack of a significant temperature effect on nesting arrival suggests that colony establishment is determined largely by habitat availability, whereas temperature influences colony size and species composition after the onset of breeding.

Wind speed had no significant effects on nesting arrival, abundance and species richness. Although strong winds can have negative effects on breeding success through nest damage, chick mortality and reduced foraging efficiency (Leberg et al. 2007; Torres et al. 2010), such effects are typically associated with extreme weather events, rather than average wind conditions. The relatively weak effect found in this study may therefore reflect the use of mean wind speed metrics that do not capture episodic storms or high wind events that can directly impact nesting colonies.

Breeding activity varied considerably among wetlands. Some sites consistently supported breeding colonies throughout the study while others experienced temporary breeding failures or large fluctuations in abundance and species richness. Such variation likely reflects differences in hydrological management, wetland connectivity, availability of nesting habitat and local disturbance regimes. Colonial waterbirds are highly mobile and often redistribute among wetlands in response to changing environmental conditions (Santoro et al. 2013; McKellar et al. 2021). Therefore, temporary breeding failures at individual wetlands may not necessarily indicate population declines, but rather redistribution within a wider network of wetlands.

While hydrological conditions were the strongest predictors of nesting arrival, colony abundance and species richness, colonial waterbird breeding dynamics are influenced by multiple interacting factors. Variables not quantified in the present study, including nesting substrate availability, vegetation structure, prey abundance, predation risk, human disturbance and wetland management practices, may also contribute to variation among wetlands. Field observations suggest that some of these factors may be important. For example, the marked decline in species richness observed at Vellode Bird Sanctuary coincided with deterioration of nesting habitat, including mortality of nesting trees and extensive coverage of remaining trees by climbing vegetation. Similarly, recent work from Melselvanoor–Kelselvanoor Bird Sanctuary reported substantial declines in breeding waterbirds associated with degradation of nesting habitat and hydrological instability (Byju et al. 2025). Similar studies elsewhere shown that habitat characteristics, nesting substrate availability, disturbance levels and vegetation structure interact with hydrological conditions to influence colonial waterbird populations (Ma et al. 2010; Rodrigues et al. 2025). These observations suggest that variation in breeding responses among wetlands cannot be explained solely by hydrological and climatic conditions.

When interpreting these results, several limitations in this study should also be considered. Climatic variables were derived from the ERA5 reanalysis dataset with a spatial resolution of approximately 0.25° (~31 km). While this resolution may not fully capture microclimatic conditions experienced within individual colonies, ERA5 combines observations from satellites, weather stations and atmospheric models to provide spatially and temporally consistent estimates of climatic conditions across large geographic regions. Since the objective of this study was to examine environmental drivers across multiple wetlands distributed across Tamil Nadu, the use of ERA5 is appropriate for identifying regional‐scale climatic influences. Nevertheless, local factors such as canopy cover, vegetation structure, wetland morphology and proximity to open water may generate colony‐scale microclimates that differ from reanalysis estimates. Future studies incorporating on‐site microclimatic measurements could provide additional insight into fine‐scale thermal conditions experienced by breeding birds.

All bird counts were performed by one trained observer during the study, which maximised consistency in detection and counting procedures across wetlands and seasons. However, this also meant that inter‐observer reliability could not be assessed independently.

Although visual delineation of open‐water areas from contemporaneous true‐colour imagery informed the selection of wetland‐specific MNDWI thresholds, no field measurements or independent reference dataset was collected specifically for accuracy assessment. Therefore, the accuracy of the satellite‐derived water masks could not be independently quantified.

Water extent was used as a surrogate for hydrological conditions and does not capture variation in water depth, hydroperiod, prey availability or water quality, all of which may influence breeding success. In addition, responses were analysed at the assemblage level, whereas species‐specific responses may vary according to differences in foraging ecology, thermal tolerance, body size and habitat requirements. Finally, habitat variables such as nesting tree availability, vegetation structure, predator abundance and anthropogenic disturbance were not quantified despite field observations suggesting their potential importance. Future studies integrating hydrological, climatic, habitat and species‐specific data would provide a more comprehensive understanding of the mechanisms governing colonial waterbird breeding dynamics.

## 5 Conservation and Management Implications

The strong influence of water extent on nesting arrival, colony abundance and species richness indicates that maintaining suitable hydrological conditions is fundamental for sustaining colonial waterbird populations. Conservation strategies should therefore prioritise the maintenance of adequate water levels during breeding seasons through effective management of reservoirs, irrigation systems and canal networks supplying water to wetlands. Similar studies worldwide have shown that restoration or maintenance of natural hydrological regimes can substantially increase breeding opportunities for colonial waterbirds (Kingsford and Johnson 1998; Scholte 2006; Brandis et al. 2018; Bino et al. 2014). Because many wetlands in Tamil Nadu depend on hydrological inputs originating beyond their immediate catchments, conservation planning should adopt a landscape‐scale perspective that considers regional water allocation and connectivity among wetlands.

Protection and restoration of nesting habitat are equally important. Conservation efforts should include protection of existing nesting trees, restoration of degraded nesting habitats and management of invasive vegetation that reduces nesting suitability. Conservation efforts should therefore include protection of existing nesting trees, restoration of degraded nesting habitats and management of invasive vegetation that reduces nesting suitability. Maintaining a network of hydrologically functional wetlands containing suitable nesting habitat will likely enhance the resilience of colonial waterbird populations under increasing climatic variability and future warming.

## Author Contributions

**Alex Jacob:** data curation (lead), formal analysis (lead), investigation (equal), methodology (equal), visualisation (lead), writing – original draft (lead). **Govindan Veeraswami Gopi:** conceptualization (lead), funding acquisition (lead), investigation (equal) methodology (equal), project administration (lead), resources (lead), validation (lead), writing – review and editing (lead).

## Funding

This work was supported by Anusandhan National Research Foundation (ANRF) under Grant No: EEQ/2021/000216.

## Conflicts of Interest

The authors declare no conflicts of interest.

## Data Availability

The data supporting the findings of this study are publicly available in the Zenodo repository at https://doi.org/10.5281/zenodo.20715976. The repository contains the environmental variables, metadata and associated files required to reproduce the results presented in this manuscript.
